# Development and validation of radiomic signature for predicting overall survival in advanced-stage cervical cancer

**DOI:** 10.3389/fnume.2023.1138552

**Published:** 2023-05-17

**Authors:** Ashish Kumar Jha, Sneha Mithun, Umeshkumar B. Sherkhane, Vinay Jaiswar, Sneha Shah, Nilendu Purandare, Kumar Prabhash, Amita Maheshwari, Sudeep Gupta, Leonard Wee, V. Rangarajan, Andre Dekker

**Affiliations:** ^1^ Department of Radiation Oncology (Maastro), GROW - School for Oncology and Reproduction, Maastricht University Medical Centre+, Maastricht, Netherlands; ^2^ Department of Nuclear Medicine, Tata Memorial Hospital, Mumbai, India; ^3^ Homi Bhabha National Institute, BARC Training School Complex, Mumbai, India; ^4^ Department of Medical Oncology, Tata Memorial Hospital, Mumbai, India; ^5^ Department of Surgical Oncology, Tata Memorial Hospital, Mumbai, India; ^6^ Advance Center for Treatment, Research, Education in Cancer, Navi-Mumbai, India

**Keywords:** cervical cancer, FIGO, prediction model, radiomics, machine learning

## Abstract

**Background:**

The role of artificial intelligence and radiomics in prediction model development in cancer has been increasing every passing day. Cervical cancer is the 4th most common cancer in women worldwide, contributing to 6.5% of all cancer types. The treatment outcome of cervical cancer patients varies and individualized prediction of disease outcome is of paramount importance.

**Purpose:**

The purpose of this study is to develop and validate the digital signature for 5-year overall survival prediction in cervical cancer using robust CT radiomic and clinical features.

**Materials and Methods:**

Pretreatment clinical features and CT radiomic features of 68 patients, who were treated with chemoradiation therapy in our hospital, were used in this study. Radiomic features were extracted using an in-house developed python script and pyradiomic package. Clinical features were selected by the recursive feature elimination technique. Whereas radiomic feature selection was performed using a multi-step process i.e., step-1: only robust radiomic features were selected based on our previous study, step-2: a hierarchical clustering was performed to eliminate feature redundancy, and step-3: recursive feature elimination was performed to select the best features for prediction model development. Four machine algorithms i.e., Logistic regression (LR), Random Forest (RF), Support vector classifier (SVC), and Gradient boosting classifier (GBC), were used to develop 24 models (six models using each algorithm) using clinical, radiomic and combined features. Models were compared based on the prediction score in the internal validation.

**Results:**

The average prediction accuracy was found to be 0.65 (95% CI: 0.60–0.70), 0.72 (95% CI: 0.63–0.81), and 0.77 (95% CI: 0.72–0.82) for clinical, radiomic, and combined models developed using four prediction algorithms respectively. The average prediction accuracy was found to be 0.69 (95% CI: 0.62–0.76), 0.79 (95% CI: 0.72–0.86), 0.71 (95% CI: 0.62–0.80), and 0.72 (95% CI: 0.66–0.78) for LR, RF, SVC and GBC models developed on three datasets respectively.

**Conclusion:**

Our study shows the promising predictive performance of a robust radiomic signature to predict 5-year overall survival in cervical cancer patients.

## 1. Introduction

Cancer is one of the most fatal diseases and is considered the second most lethal disease across the world (1). As per Global Cancer Statistics 2020 (GLOBOCAN 2020), cervical cancer is the 4th commonest cancer worldwide, 6th commonest cancer in developed countries and 2nd commonest cancer in developing countries in the female population (2, 3). The cervical cancer-related mortality rate among women varies across the globe and there is a distinct difference in developed and developing countries (2–4). Breast and cervical cancer are the leading causes of cancer death in 103 and 42 countries, respectively, whereas lung cancer is the leading cause of cancer death in 28 countries (1–4). Cervical cancer management has been approached on two fronts i.e., prevention or early detection of cervical cancer by implementing screening programs and treatment of cervical cancer using evidence-based medicine (8–14). The incidence of cervical cancer in developed countries has reduced to half between 1972 and 2018 (8–10). The reason for the reduced incidence and mortality rate can be attributed to the effective implementation of cervical cancer screening and HPV vaccination programs. Availability of several new technologies or advancements in existing technology like CT, PET/CT, ultrasound and MRI has led to early diagnosis and better staging of the disease, leading to improvement in overall survival and quality of life index (11–14). The staging of cervical cancer is very complex and technically demanding. The staging system developed by the International Federation of Obstetrics and Gynecology (Fédération Internationale de Gynecologie et d’Obstetrique, or FIGO) is used for cervical cancer. Bhatla N. et.al. have published the recently revised FIGO staging of carcinoma of the cervix uteri to differentiate the various stages and substages of the disease (15). Improvement in diagnostic accuracy due to the implementation of newer technologies like PET/CT, MRI, and transvaginal ultrasound has improved cervical cancer staging and treatment in the last few years. As conventional treatment has a very low response rate of around 20–30 per cent, it proves that the “one-size-fits-all” principle usually doesn't work in cancer management (15–17). In the last few years, diagnostic modalities like immunohistochemistry (IHC), genetic profiling, and tumor marker studies have established the fact that there are variations in disease in the same disease in different patients (18). Hence, cancer treatment is gradually shifting towards personalized treatment or tailored treatment and replacing conventional treatment (19). With the growing use of various computer-aided technologies in oncology in the last decade, these technologies have taken the forefront in cancer management worldwide (20). These technologies are being utilized for diagnosis, treatment planning, interim evaluation, and follow-up of the disease. In the last few years, as the effort is being taken to provide personalized treatment to the patients, the ability of these technologies is being tested to predict treatment outcome, toxicity profile, and treatment selection for patients. Utilization of available technologies like machine learning, radiomics, genomics, etc. for enabling personalized treatment, especially for those at high risk and who are responding very poorly to standard treatment protocols, is of great interest for clinicians (20). Such a technological-driven system has shown promising results in the selection or modification of treatment plans, to improve the treatment outcome (21–23). Major types of ML techniques, including Decision Tree (DT), Support Vector Machine (SVM), Artificial Neural Networks (ANN), Naïve Bayesian Classifier (BC), Bayesian Network (BN), K-Nearest Neighbor (KNN) and Random Forest (RF), have been used for nearly three decades in cancer detection (21–25). In cancer prediction modelling, the main three predictive tasks are the prediction of cancer susceptibility, the prediction of cancer recurrence/metastasis, and the prediction of survival. Several such technology-driven prediction models have been developed, tested, and utilized in the last decade in screening programs and the treatment of cervical cancer (26–39). However, several prediction models have been developed using clinical and radiomics features predicting survival outcomes but the stability of radiomic features has been questioned by many researchers. In our earlier study, we have performed a detailed stability study of CT radiomic features and found around 100 robust radiomic features. In this study, we have tried to find the prediction capability of robust radiomic features with and without clinical features in predicting 5-year overall survival. This study is also the first of this kind from India.

## 2. Materials and method

### 2.1. Patient demographics

The study was approved by the institutional ethics committee as a retrospective study with a waiver of consent. In total 68 patients were included in this study and had ages ranging 45–72 years (median: 56 years), at the time of diagnosis. All patients diagnosed with cervical cancer between 2005 and 2009 and who were treated with definitive chemoradiotherapy or concomitant chemo and radiation therapy were included in this study. External beam radiation therapy (EBRT) dose range between 43.2 and 60.4 Gy (median = 50 Gy) was considered as radiotherapy procedures. Disease staging was performed according to the International Federation of Gynecology and Obstetrics (FIGO) classification. The numbers of patients in various FIGO stages in this cohort of patients are provided in Table 1. The majority of the patients (85%) had squamous cell carcinoma and only a few patients (15%) had other histologies. From diagnosis to the last follow-up, the meantime was 72 (range: 5–140) months. 48 of the 68 patients had survived more than five years, whereas 26 had survived less than five years. In our study, we have aimed to establish the correlation between radiomics/clinical features and overall survival. The initial characteristics of the study population are given in Table 1.

**Table 1:** Demographic details of the study population.

Characteristics	Patients
Sample size	68
Age (years)	56 (Range: 45–72)
**Sex**	
Male	0
Female	68/68 (100%)
**Tumor type**	
Cervix cancer	68/68 (100%)
**FIGO stages**	
Stage 3	20
Stage 4	48
**Pelvic Node**	
Yes	42
No	26
**Retroperitoneal Node**	
Yes	58
No	10
**Surgery**	
Yes	15
No	53
**Overall survival (OS)**	
>5-year	42
<5-year	26

20 clinical, pathological and radiological features were extracted from electronic health records as approved by the hospital ethics committee; out of that, 13 features were used for further processing. Pretreatment PET/CT scans were also downloaded from the PACS for radiomic extraction. 1,093 CT radiomics features were extracted from the CT series of PET/CT scans.

### 2.2. PET/CT imaging procedure

All of the baseline PET/CT scans were performed using Gemini TF16 or Gemini TF64 PET/CT scanners (Philips Medical Systems, Netherlands) (40). F-18 FDG radiopharmaceutical was administered to the patient as per institutional protocol i.e., 4–5 MBq/kg body weight after 6 h of fasting. Scans were performed between 60 min and 100 min after administration of the radiopharmaceutical.

Contrast-enhanced CT scans were performed after the injection of 60–80 ml of non-ionic contrast using the protocol mentioned in Supplementary Table S1. CT images were reconstructed using the Filtered back project (FBP) reconstruction algorithm.

### 2.3. Radiomic extraction

DICOM images of PET/CT scan were downloaded on Philips Intellispace Discovery (research-only build; Philips Medical System, Eindhoven, The Netherlands) from PACS. The tumor was contoured using 3D contouring software installed on Intellispace Discovery by a 15-year experienced medical physicist and checked & approved by a 30-year experienced nuclear medicine physician. The contours were saved as RTStructure by the name of GTV. Subsequently, the image and GTV were transferred to the research computer for radiomic extraction.

Images and GTV were converted into NRRD format using Plastmatch software (41). Thereafter, pre-processing steps were applied using an in-house developed python script and the Pyradiomics package (42) for radiomic extraction. Resampling: Images were resampled using a 2 × 2 × 2 mm cube isotropic voxel. Filtering and transformation of image: From the original images, three sets of filtered images were produced applying Laplacian of Gaussian (LoG) filters with 1, 2, and 3 mm sigma values. We also generated 8 sets of wavelet-transformed images using eight combinations of high-pass and low-pass wavelet filters (42–44).

A total of 1,093 radiomic features were extracted from 12 sets of images (1 set of original images, 3 sets of LoG images, and 8 sets of Wavelet Images) and corresponding GTVs (42).

### 2.4. Prediction algorithm used

The commonly used machine learning algorithms for classification problems i.e., Logistic regression (LR), Random Forest classifier (RF), Gradient boosting classifier (GBC), and Support vector classifier (SVC), were used for prediction model development (45–52).

### 2.5. Feature selection

**Figure 1:**
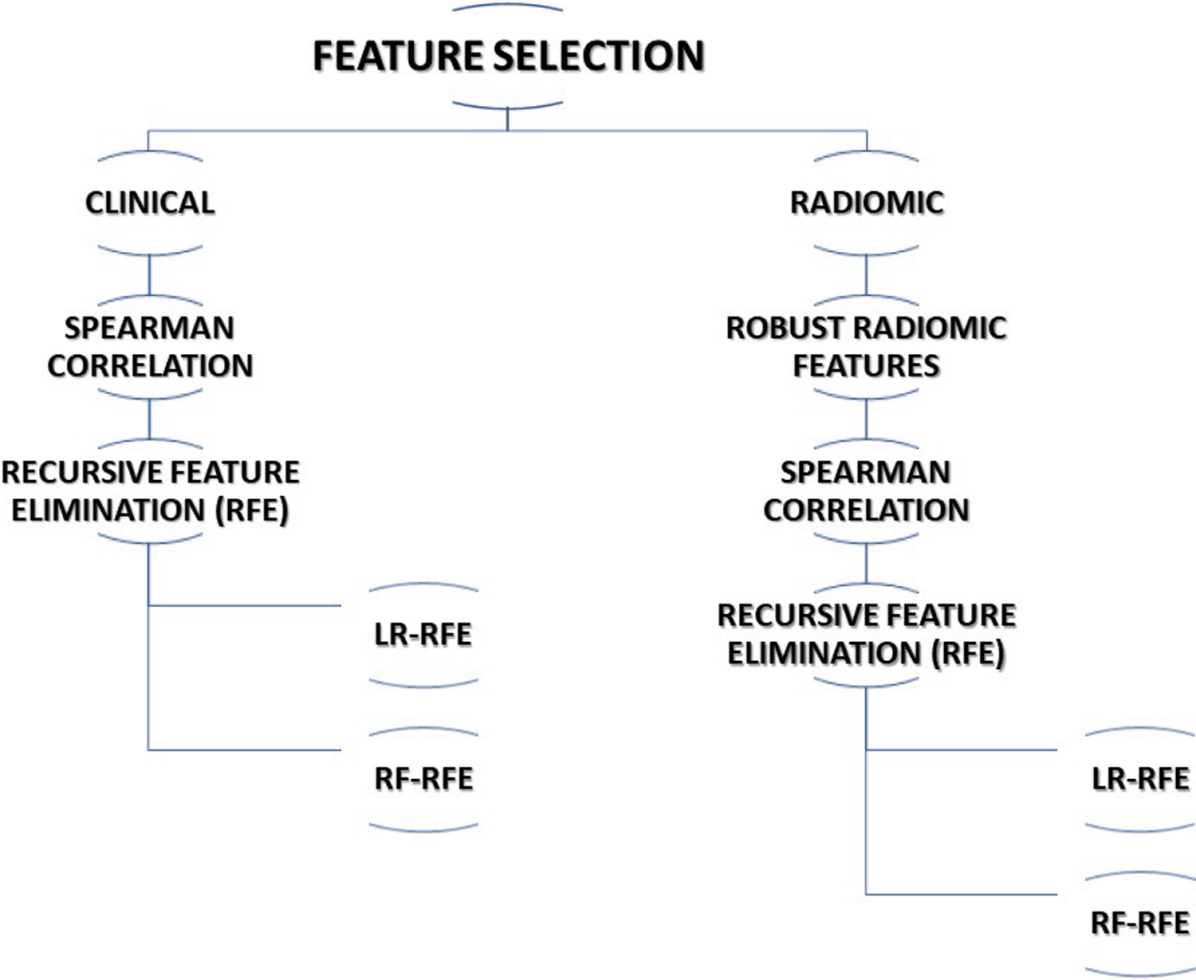
Feature selection algorithm used in this study.

The multi-step process was adopted for feature selection in this study. The following subsections describe the various methods adopted for feature selection. The steps utilized for feature selection are summarized in Figure 1.

#### 2.5.1. Clinical features selection

Considering the completeness of data, 13 clinical features were selected for further processing. Spearman correlation test was performed to find correlating features and reduce the redundancy among the features. The association of clinical features with outcome i.e., 5-years overall survival (OS) was carried out using a t-test. Finally, recursive feature elimination (RFE) methods using logistic regression (RFE-LR) and random forest (RFE-RF) were applied to select two sets of features for prediction model development.

#### 2.5.2. Radiomic feature selection

We opted for a two-step process to select the best radiomic features for OS prediction out of 1,093 radiomic features extracted from CT images. In the first step of feature selection, we included 121 stable radiomic features for the next step of feature selection based on our earlier radiomic stability study (53). In the second step of feature selection, we performed a Spearman correlation test to identify redundant features. In step 3, recursive feature elimination (RFE) methods using logistic regression (RFE-LR) and random forest (RFE-RF) were applied to select two sets of features for the prediction model development.

#### 2.5.3. Combined (clinical + radiomic) features selection

The top 7 clinical features and top 15 radiomic features that were identified in clinical and radiomic feature selection steps were used to select the best features for the combined model. Recursive features selection (RFE) methods using logistic regression (RFE-LR) and random forest (RFE-RF) were applied to select two sets of features for prediction model development.

Features selected using random forest model were used to develop models using random forest (RF) Support vector classifier (SVC) and Gradient Boosting and features selected using logistic regression (LR) were used to develop the logistic regression model.

### 2.6. Nested cross-validation

Nested cross-validation was performed on the entire dataset using 7 outer and 6 inner loops for tuning the hyperparameters of the models (54). Finally, a random train-test split (in 7:3 ratio) of data was performed and a prediction model was developed and validated.

### 2.7. Data balancing

After the train-test split, the training dataset was used to develop the prediction models with and without balancing the train data set for survival outcomes. Data balancing was performed by using minority oversampling. Validation was performed using the test data set without balancing the data.

### 2.8. Model development

**Figure 2:**
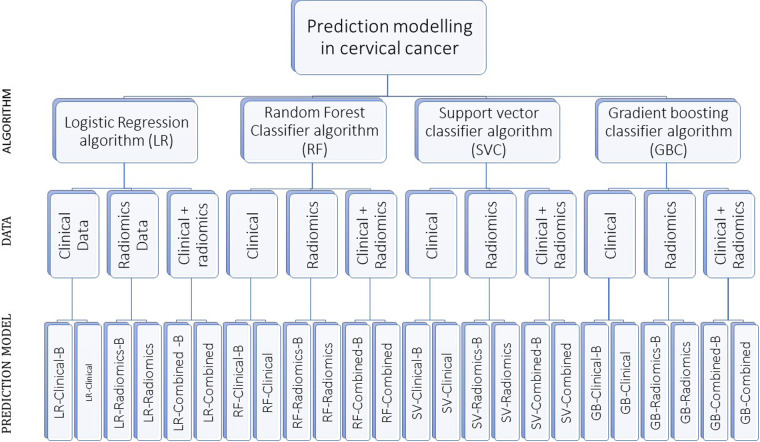
The figure shows our algorithm to develop 24 prediction models using various combinations. “-B” in the model’s name indicates the model developed using a balanced train data set.

A total of 24 prediction models were developed using the aforementioned four prediction algorithms, three data sets with and without balancing the train data sets (Supplementary Table S2 and Figure 2).

### 2.9. Model evaluation and selection

All the developed models were evaluated by plotting the area under the receiver operator curve (AUC) to graphically represent the association between the features and the outcome i.e., 5-year overall survival in the validation set. The best model was selected based on the performance score of each model in the validation set.

### 2.10. Statistical analysis

Statistical analyses were performed using R (v3.5.2, the R foundation for statistical computing, Vienna, Austria) or Python 3.9.0 software. Prediction model development and validation of models were performed using python 3.9.0 software.

## 3. Results

**Figure 3:**
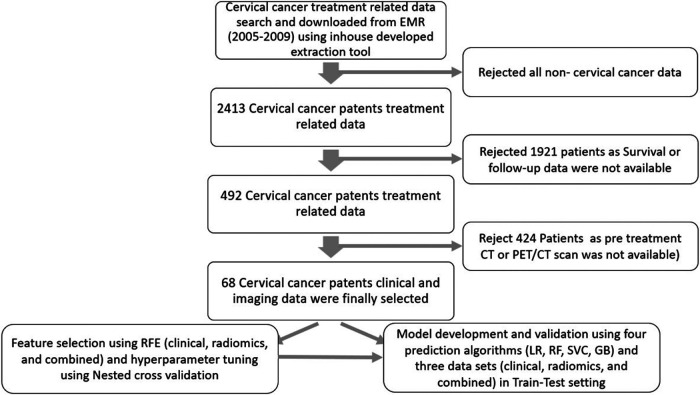
The figure shows the process of data collection and prediction model development.

In total, 68 patients who fulfilled the criteria of completeness of data sets were selected for this study. The details of data collection are provided in Figure 3.

### 3.1. Feature selection

#### 3.1.1. Clinical

**Figure 4:**
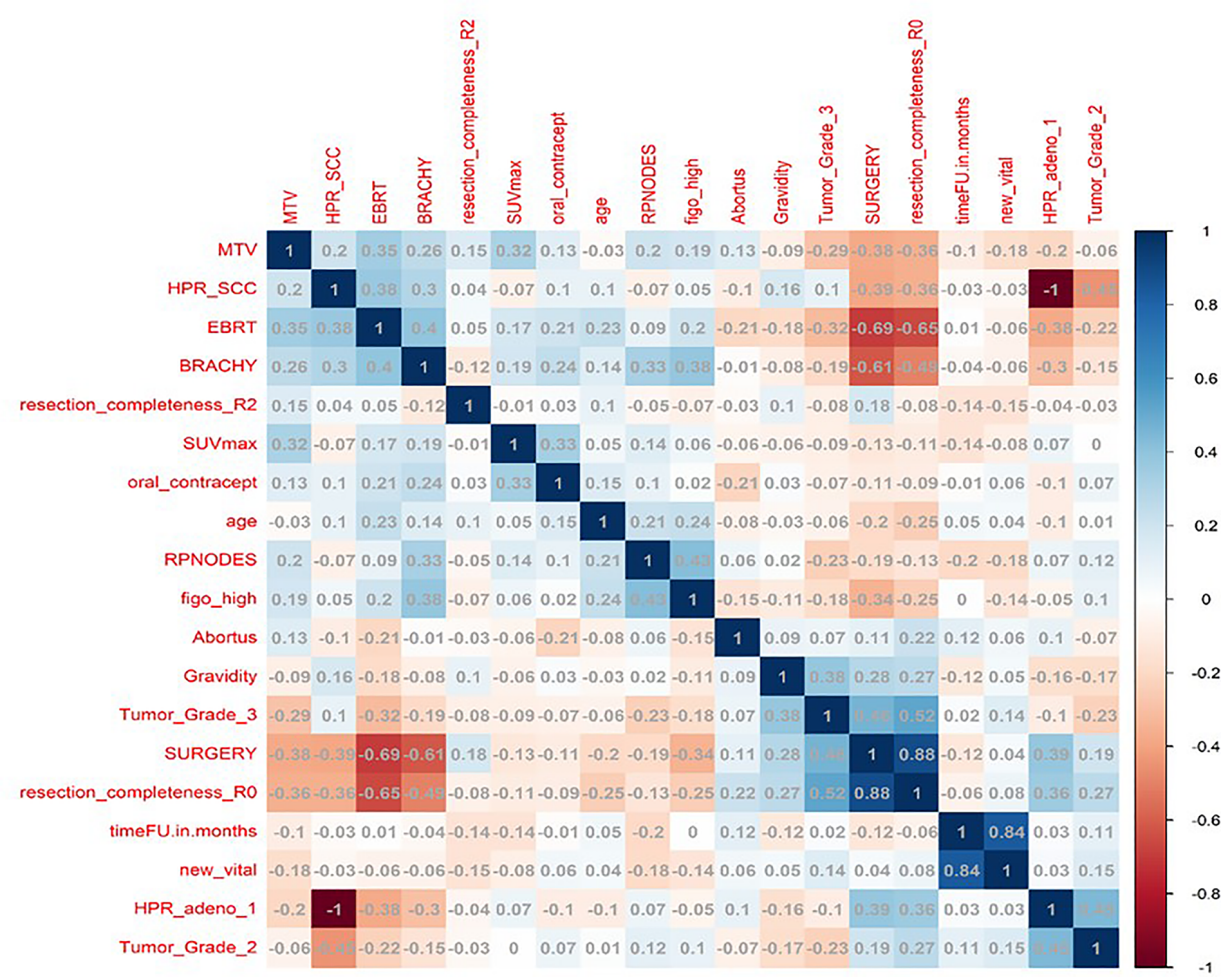
The figure shows the spearman correlation among the clinical features.

In total, 13 clinical and radiological features were used for this study. Figure 4 shows the Spearman correlation among the features; there are a few features which have a strong positive and a few which have a strong negative correlation. For example, surgery and *R*0 resection have a strong negative correlation with EBRT and Brachytherapy (*r*2 = −0.65 to −0.69) HPR adenocarcinoma has a very strong negative correlation (*r*2 = −1) with that of HPR squamous cell carcinoma; they probably do not exist together and cannot both be used as they are redundant features. Whereas follow-up time in months has a strong positive correlation (*r*2 = 0.8) with that of new vital (recurrence), which may be because increasing follow-up time increases the chance of recurrence. Surgery has a strong positive correlation (*r*2 = 0.88) with that of *R*0 resection and probably both cooccur. Among strong correlating features, one feature each was selected for the next step of feature selection. Recursive feature elimination (RFE) was performed using logistic regression and random forest algorithms. A total of 5 clinical features were found to be significant for each algorithm independently (Table 2 and Figure 6).

**Table 2:** The table shows the number of features selected, accuracy and kappa value for various combinations of data sets using multivariate recursive feature elimination with logistic regression and random forest.

Feature selection technique	Feature type	Number of features selected	Accuracy with selected features	Kappa value
Recursive Feature Elimination with Logistic regression	Clinical	5	0.69	0.31
	Radiomics	3	0.64	0.17
	Clinical + Radiomics	5	0.68	0.26
Recursive Feature Elimination with Random Forest	Clinical	5	0.68	0.32
	Radiomics	4	0.72	0.38
	Clinical + Radiomics	5	0.77	0.46

#### 3.1.2. Radiomics

**Figure 5:**
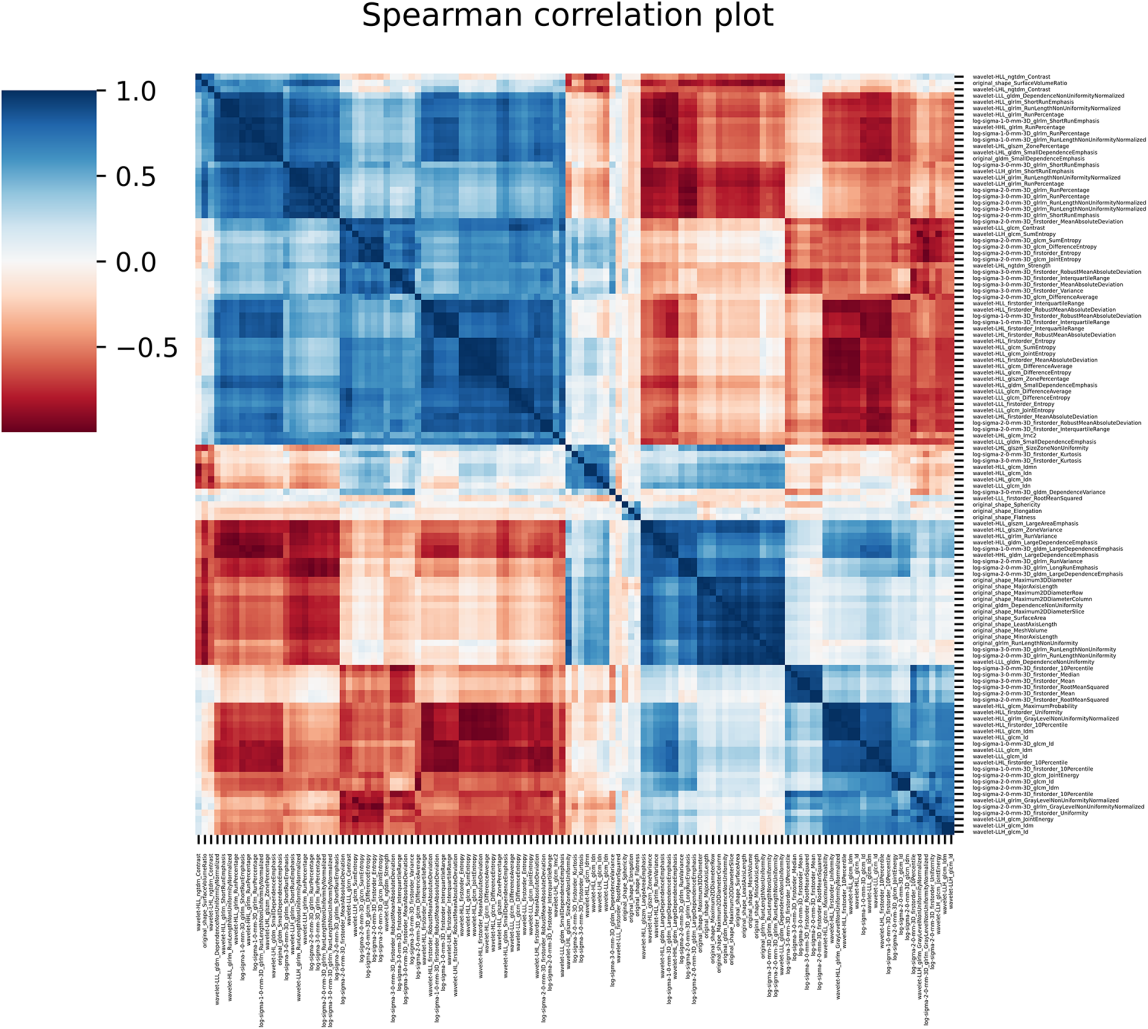
The figure shows the spearman correlation among the radiomic features showing clusters of features with positive and negative correlations.

**Figure 6:**
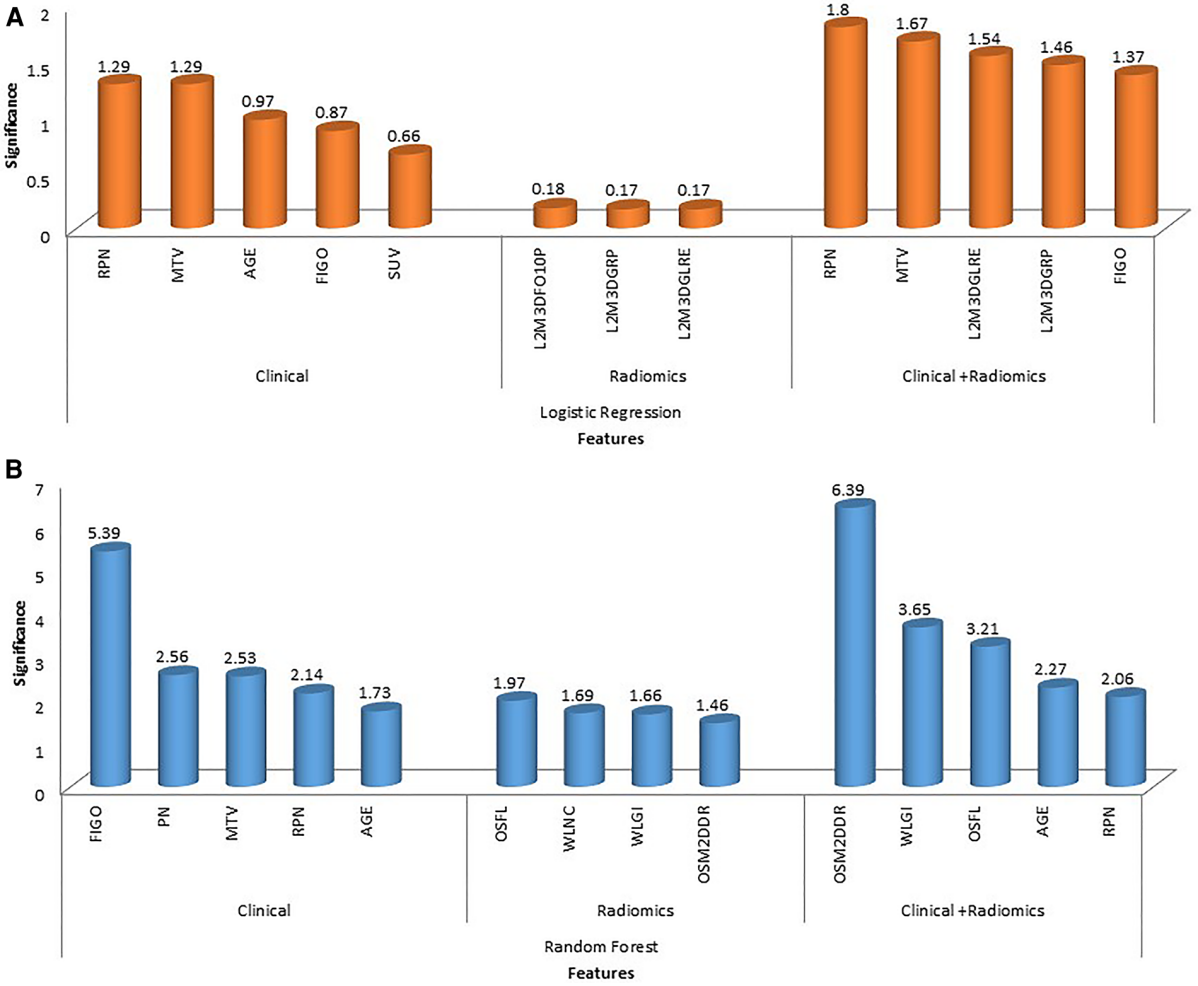
The figure shows feature importance in various combinations of algorithms and features. The first row shows feature importance using a logistic regression algorithm for clinical, radiomic and combined (clinical + radiomics) features (**A**); the second row shows feature importance using random forest algorithm for clinical, radiomic and combined (clinical + radiomics) features (**B**). (Abbreviations: OSFL, original_shape_flatness; WLNC, wavelet_LHL_ngtdm_contrast; WLGI, wavelet_LLL_glcm_Idn; OSM2DDR, original_shape_maximum2DDiameterRow; L3M3DFO10P, log-sigma-3-0-mm-3D_firstorder_10percentile; L2M3DGRP, log-sigma-2-0-mm-3D_glrlm_runPercentage; L2M3DGLFE, log-sigma-2-0-mm-3D_glrlm_longRunEmphasis).

121 stable radiomics features based on our earlier study were included in this study (53). Spearman correlation shows 10 distinct clusters (Figure 5) and these clusters had positive or negative correlations. Based on these clusters and *r*2 value, 15 radiomic features were selected to include in the next step of feature selection. Recursive feature elimination (RFE) was performed using logistic regression and random forest algorithms. In total, 3 and 4 radiomic features were found to be significant for logistic regression and random forest algorithms respectively (Table 2 and Figure 6).

#### 3.1.3. Combined (clinical + radiomics)

Among clinical and radiomic features selected independently, the most significant mixed features were selected using recursive feature elimination with logistic regression and random forest algorithms. In total, 5 clinical + radiomics features were found to be significant for each of the algorithms separately (Table 2 and Figure 6). The selected radiomic feature shows the distinct distribution of feature values in two groups of patients i.e., OS > 5 years and OS < 5 years. The box plots show the distribution of all the selected features in two groups of patients (Supplementary Figures S1–S9).

### 3.2. Model development and validation

Four algorithms i.e., Logistic regression (LR), Random Forest (RF), Support vector classifier (SVC) and gradient boost classifier (GBC), were used for prediction model development. There were a total of 24 prediction models using four prediction algorithms for clinical, radiomics and combined features.

Nested cross-validation: Nested cross-validation was performed for all the prediction algorithms for tuning their hyperparameters. The prediction algorithms along with the best hyperparameters and validation scores are shown in Table 3.

**Table 3:** This table shows the selected hyperparameters and nested cross-validation scores of various models.

Algorithms	Features	Hyperparameters	Accuracy in nested cross-validation
Logistic regression	Clinical	{c: “10”, penalty: “12”, solver: “newton-cg”}	0.66 (±0.17)
	Radiomics	{c: “10”, penalty: “12”, solver: “liblinear”}	0.68 (±0.09)
	Clinical + Radiomics	{c: “100”, penalty: “12”, solver: “newton-cg”}	0.66 (±0.06)
Random forest	Clinical	{bootstrap: “true”, criterion: “gini”, max_depth: “10”, “min_samples_leaf”: 2,n_estimators: “80”}	0.66 (±0.15)
	Radiomics	{“bootstrap”: True, “criterion”: “gini”, “max_depth”: 25, “min_samples_leaf”: 2, “n_estimators”: 40}	0.79 (±0.09)
	Clinical + Radiomics	{bootstrap: “true”, criterion: “gini”, max_depth: “10”, “min_samples_leaf”: 2,n_estimators: “80”}	0.75 (±0.07)
Support vector classifier	Clinical	{c: “1”, gamma: “1”, kernel: “linear”}	0.72 (±0.11)
	Radiomics	{c: “0.1”, gamma: “0.001”, kernel: “rbf”}	0.74 (±0.08)
	Clinical + Radiomics	{c: “100”, gamma: “0.001”, kernel: “rbf”}	0.67 (±0.16)
Gradient boost classifier	Clinical	{learning_rate: “0.1”, max_depth: “7”, n_estimators: “60”}	0.75 (±0.12)
	Radiomics	{“learning_rate”: 0.1, “max_depth”: 7, “n_estimators”: 80}	0.75 (±0.13)
	Clinical + Radiomics	{learning_rate: “1”, max_depth: “3”, n_estimators: “10”}	0.75 (±0.09)

**Figure 7:**
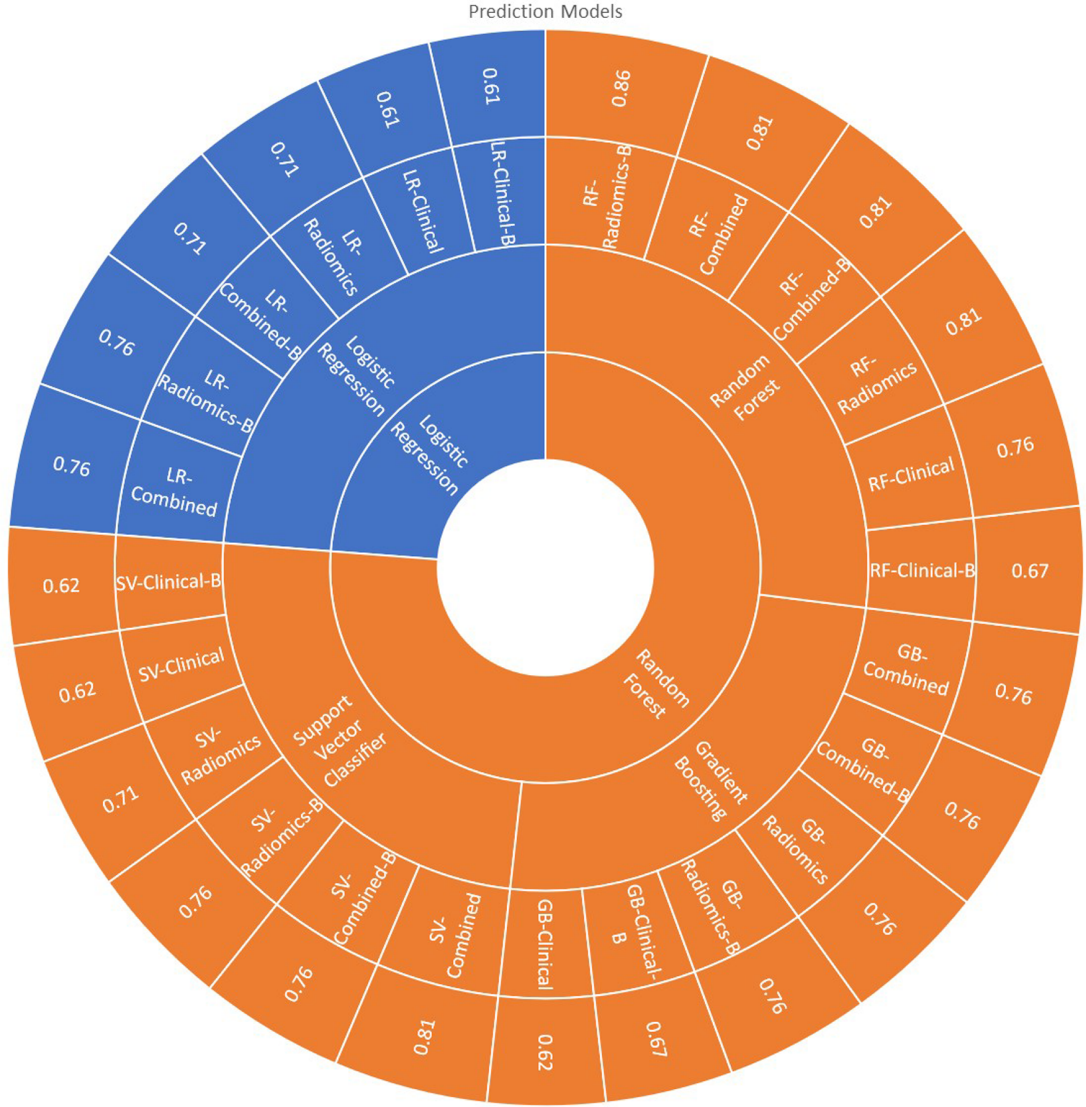
The figure shows the prediction models with prediction accuracy in the validation set.

All 24 models showed good prediction capability of 5-year overall survival. The average accuracy and AUC in validation sets across all the 24-prediction models were found to be 0.73 (95% CI: 0.66–0.80) and 0.60 (95% CI: 0.49–0.71) respectively. The detailed complete validation scores of all the models are shown in Table 4 and Figure 7.

**Table 4:** The table shows accuracy, PPV, NPV, F1-score and AUC of all the models.

Feature selection function	ML algorithm	Prediction model	Accuracy	Precision	Recall	F1-Score	AUCh
Logistic regression	Logistic Regression	LR-Clinical-B	0.61	0.63	0.62	0.62	0.65
		LR-Clinical	0.61	0.63	0.62	0.6	0.65
		LR-Radiomics-B	0.76	0.83	0.76	0.78	0.60
		LR-Radiomics	0.71	0.77	0.71	0.73	0.62
		LR-Combined-B	0.71	0.77	0.71	0.73	0.56
		LR-Combined	0.76	0.78	0.76	0.77	0.51
Random forest	Random Forest	RF-Clinical-B	0.67	0.7	0.67	0.67	0.65
		RF-Clinical	0.76	0.77	0.76	0.76	0.71
		RF-Radiomics-B	0.86	0.86	0.86	0.85	0.82
		RF-Radiomics	0.81	0.81	0.81	0.81	0.81
		RF-Combined-B	0.81	0.81	0.81	0.81	0.70
		RF-Combined	0.81	0.78	0.81	0.78	0.71
	Support Vector Classifier	SV-Clinical-B	0.62	0.38	0.62	0.47	0.39
		SV-Clinical	0.62	0.38	0.62	0.47	0.59
		SV-Radiomics-B	0.76	0.76	0.76	0.76	0.70
		SV-Radiomics	0.71	0.74	0.71	0.69	0.83
		SV-Combined-B	0.76	0.83	0.76	0.78	0.82
		SV-Combined	0.81	0.85	0.81	0.82	0.82
	Gradient Boosting	GB-Clinical-B	0.67	0.66	0.67	0.66	0.68
		GB-Clinical	0.62	0.6	0.62	0.61	0.68
		GB-Radiomics-B	0.76	0.83	0.76	0.78	0.74
		GB-Radiomics	0.76	0.83	0.76	0.78	0.82
		GB-Combined-B	0.76	0.78	0.76	0.77	0.74
		GB-Combined	0.76	0.74	0.76	0.75	0.72

#### 3.2.1. Logistic regression model

The average accuracy and AUC for logistic regression models across six models developed with various combinations were found to be 0.69 (95% CI: 0.62–0.76) and 0.60 (95% CI: 0.55–0.65) respectively. The AUC of all the logistic regression models is shown in Supplementary Figure S10. Radiomics [accuracy: 0.76 (LR-Radiomics-B); 0.71 (LR-Radiomics)] or combined prediction [accuracy: 0.71 (LR-Combined-B); 0.76 (LR-Combined)] models had better prediction capabilities in comparison to clinical models [accuracy: 0.61 (LR-Clinical-B); 0.61 (LR-Clinical)] developed with logistic regression algorithm.

#### 3.2.2. Random forest model

The average accuracy and AUC for random forest models were found to be 0.79 (95% CI: 0.72–0.86) and 0.73 (95% CI: 0.66–0.80) respectively. The AUC of all the Random Forest models is shown in Figure 7. Radiomics [accuracy: 0.86 (RF-Radiomics-B); 0.81 (RF-Radiomics)] or combined prediction [accuracy: 0.81 (RF-Combined-B); 0.81 (RF-Combined)] models had better prediction capabilities in comparison to clinical models [accuracy: 0.67 (RF-Clinical-B); 0.76 (RF-Clinical)] developed with random forest algorithm.

#### 3.2.3. Support vector classifier (SVC) model

The average accuracy and AUC for support vector models were found to be 0.71 (95% CI: 0.63–0.79) and 0.69 (95% CI: 0.51–0.87) respectively. The AUC of all the support vector classifier models is shown in Supplementary Figure S12. Radiomics [accuracy: 0.76 (SV-Radiomics-B); 0.71 (SV-Radiomics)] or combined prediction [accuracy: 0.76 (SV-Combined-B); 0.81 (SV-Combined)] models had better prediction capabilities in comparison to clinical models [accuracy: 0.62 (SV-Clinical-B); 0.62 (SV-Clinical)] developed with support vector classifier algorithm.

#### 3.2.4. Gradient boosting classifier (GBC) model

The average accuracy and AUC for gradient boosting models were found to be 0.72 (95% CI: 0.66–0.78) and 0.73 (95% CI: 0.68–0.78) respectively. The AUC of all the Gradient busting classifier models is shown in Supplementary Figure S13. Radiomics [accuracy: 0.76 (GB-Radiomics-B); 0.76 (GB-Radiomics)] or combined prediction [accuracy: 0.76 (GB-Combined-B); 0.76 (GB-Combined)] models had better prediction capabilities in comparison to clinical models [accuracy: 0.67 (GB-Clinical-B); 0.62 (GB-Clinical)] developed with gradient boosting algorithm.

### 3.3. Model selection

RF-Radiomics-B model had the best prediction accuracy (accuracy = 0.86; AUC = 0.82) among all 24 models developed. The average prediction accuracy for clinical, radiomic, and combined models were found to be 0.65 (95% CI: 0.60–0.70), 0.72 (95% CI: 0.63–0.81) and 0.77 (95% CI: 0.72–0.82) respectively. The average prediction accuracy for logistic regression, random forest, support vector classifier, and gradient boosting classifier models were found to be 0.69 (95% CI: 0.62–0.76), 0.79 (95% CI: 0.72–0.86), 0.71 (95% CI: 0.62–0.80), and 0.72 (95% CI: 0.66–0.78) respectively.

## 4. Discussion

Our study shows the significance of radiomic features in generating statistical machine-learning models for disease outcomes like 5-year overall survival prediction in cervical cancer. With this study, we were able to identify the gap in the data archival system in our hospital related to medical image archives as well as other clinical data points as described in the results section. With this study, we were able to determine the most effective radiomic feature and their combination for the prediction of disease outcomes. A rigorous method of feature selection by applying various techniques has helped this study to select the most efficient features which can become a digital signature for the stated disease outcome. We tested various prediction algorithms with radiomics and clinical features separately and in combination. In multivariate analysis with random forest, radiomic features were found to be better associated with disease outcomes in our cohort. Our result was consistent with various other studies performed on cervical cancer outcome prediction. If we consider our study with other studies performed in this field, our study design had similarities with others, although we tested several prediction algorithms to select the best fits for our cohort. Our finding is consistent with other similar studies performed earlier (12, 14, 15, 26–39, 55–66). Clinical features like age, presence or absence of retroperitoneal node, and peritoneal node FIGO stage at the time of diagnosis were also found to be prognostic markers in our study which was consistent with the published literature (12, 14, 15, 26–33, 57–60). In univariate and multivariate analysis clinical features i.e., Age, FIGO stage, absence and presence of retroperitoneal node and peritoneal node, and imaging features i.e., SUV MTV found an association with 5-year overall survival, which was consistent with other published literature (12, 14, 29, 33, 34, 57, 61, 66). Similarly in univariate and multivariate studies, radiomic features showed a significant association with 5-year OS which is also consistent with published literature (28, 29, 34, 66). As we had selected only stable radiomic features based on our earlier study (53), this shows the repeatable and reproducible radiomic features also show excellent prognostic and predictive value in cervical cancer. The effort of the radiomic community should be to identify the robust features and find out the predictive capabilities of those stable features in various disease groups for various prediction endpoints. Among various prediction models tested in our study, RF-Radiomics-B random forest model showed the best accuracy in nested cross-validation and the train-test final model outperformed all the prediction models used in our study. Whereas LR-Clinical-B and LR-Clinical logistic regression models showed the lowest accuracy in predicting overall survival in this study. When we compared the performance score of prediction models with radiomic, clinical and combined models, again random forest and gradient boosting models were at the top.

The average accuracy of clinical models with all four prediction algorithms was less than that of radiomics and combined models, which is similar to previously published work (26–30). The radiomic and combined model performance across all four prediction algorithms were found to be more or less similar. Our study also confirms the superiority of radiomic features over clinical features in predicting overall survival in cervical cancer. Comparing the prediction algorithms, the random forest-based prediction models had better accuracy in comparison to the other three which affirms the findings of earlier published literature in cervical cancer (26, 27). We found little difference between the models developed with or without balanced train sets, perhaps because the event rate in our study was adequately balanced and balancing was not required as an additional step. The radiomic community has been concerned about the stability of radiomic features and is skeptical about stable radiomic features' ability to predict outcomes (67). This is probably the first study published on cancer prediction modelling using stable radiomic features independently or in combination with clinical features. In our study, we were able to show that radiomic features can be used for 5-year overall prediction in cervical cancer. This was also the first prediction modelling study to be conducted on cervical cancer patients in India. Other researchers in India will be motivated to conduct prediction modelling studies for evolving digital signatures of disease outcomes based on our study. This study was a single-center study with a small sample size and no external or prospective validation, which limits the study somewhat. The future will involve repeating this study at our hospital with a larger sample size, as well as initiating multicentric studies to develop a universally accepted model. It is the ultimate objective of this research to validate this model using prospective clinical trials and then implement decision support systems in clinics based on a validated predictive model with retrospective and prospective data.

## 5. Conclusion

We have demonstrated in our study that robust radiomic features are predictive of 5-year overall survival for cervical cancer patients. According to this study, random forest prediction algorithms can predict better than other algorithms. The model's predictive ability is slightly improved by using data balancing. Although radiomic features are superior to clinical features in terms of prediction abilities, they are most effective when combined with clinical features. Overall, this study suggests the importance of radiomics and artificial intelligence in implementing decision-support systems in the management of cervical cancer.

## Data Availability

The raw data supporting the conclusions of this article will be made available by the authors, without undue reservation.
