# Supplementary material for: Development and validation of radiomic signature for predicting overall survival in advanced-stage cervical cancer
Source: Front Nucl Med. 2023 May 17;3:1138552. doi: 10.3389/fnume.2023.1138552 (PMC11440856; doi:10.3389/fnume.2023.1138552)
Supplement: Supplementary file 1 [file Datasheet1.docx]

Supplementary Material

# Supplementary Table

| Tube Voltage (kVp) | Tube current (mA) | Slice thickness (millimeters) | Pitch (millimeters) | Voxel size (cubic millimeters) | Reconstruction Technique |
| --- | --- | --- | --- | --- | --- |
| 120 | 100-200 Auto-mA | 3.75 | 3.75 | 1.17 × 1.17 × 3.75 | Filtered back project (FBP) |

Supplementary Table 1: Overview of CT imaging protocol

| **ML algorithm** | **Feature used** | **Train data balancing** | **Prediction Model** |
| --- | --- | --- | --- |
| Random Forest | Clinical | With | RF-Clinical-B |
|  | Clinical | Without | RF-Clinical |
|  | Radiomics | With | RF-Radiomics-B |
|  | Radiomics | Without | RF-Radiomics |
|  | Clinical + Radiomics | With | RF-Combined-B |
|  | Clinical + Radiomics | Without | RF-Combined |
| Gradient Boosting | Clinical | With | GB-Clinical-B |
|  | Clinical | Without | GB-Clinical |
|  | Radiomics | With | GB-Radiomics-B |
|  | Radiomics | Without | GB-Radiomics |
|  | Clinical + Radiomics | With | GB-Combined-B |
|  | Clinical + Radiomics | Without | GB-Combined |
| Support Vector Classifier | Clinical | With | SV-Clinical-B |
|  | Clinical | Without | SV-Clinical |
|  | Radiomics | With | SV-Radiomics-B |
|  | Radiomics | Without | SV-Radiomics |
|  | Clinical + Radiomics | With | SV-Combined-B |
|  | Clinical + Radiomics | Without | SV-Combined |
| Logistic Regression | Clinical | With | LR-Clinical-B |
|  | Clinical | Without | LR-Clinical |
|  | Radiomics | With | LR-Radiomics-B |
|  | Radiomics | Without | LR-Radiomics |
|  | Clinical + Radiomics | With | LR-Combined-B |
|  | Clinical + Radiomics | Without | LR-Combined |

Supplementary Table: 2: the table shows the prediction models developed in various combination

# Supplementary Figures

The following box plots show the distribution of different radiomic features, mtv and age in the two groups of patients with overall survival > 5 years and < 5 years.


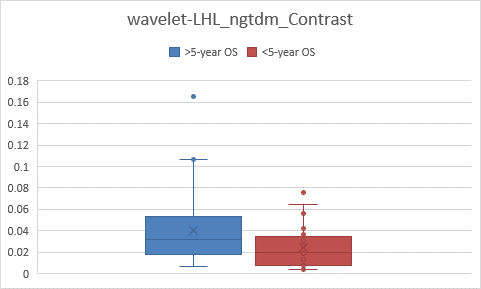


Supplementary figure 1: figure shows distinct distribution of Wavelet-LHL-NGTDM-Contrast feature patients having survival > 5 years and < 5 years.


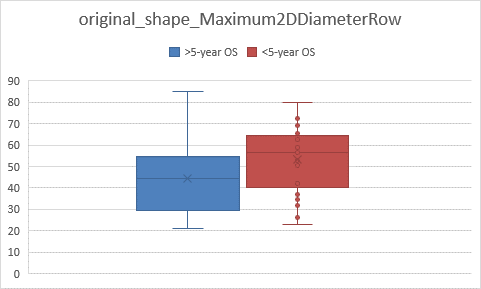


Supplementary figure 2: Figure shows distinct distribution of Original-Shape-Maximum2DDiameterRow feature in patients having survival > 5 years and < 5 years.


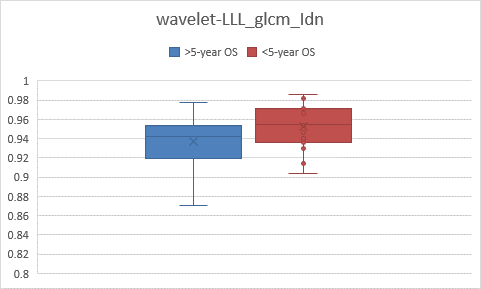


Supplementary figure 3: figure shows distinct distribution of Wavelet-LLL-GLCM-Idn feature in patients having survival > 5 years and < 5 years.


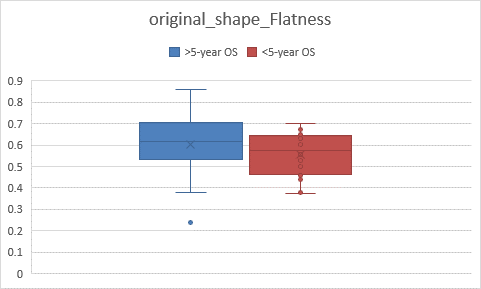


Supplementary figure 4: figure shows distinct distribution of Original-Shape-Flatness feature in patients having survival > 5 years and < 5 years.


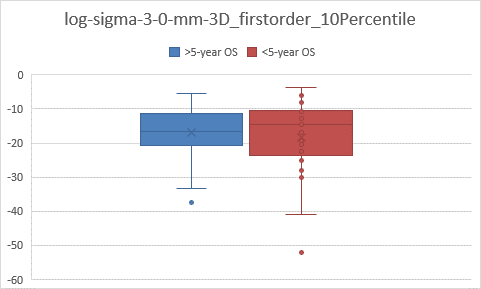


Supplementary figure 5: figure shows distinct distribution of LOG-sigma-3.o-mm-3D-firstorder-10Percentile feature in patients having survival > 5 years and < 5 years.


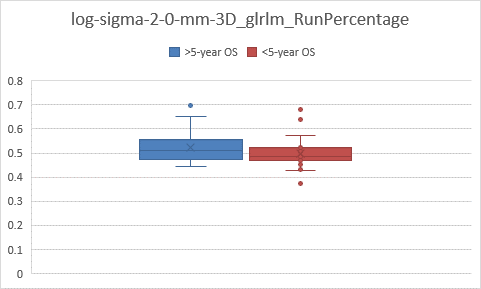


Supplementary figure 6: figure shows distinct distribution of LOG-sigma-2.o-mm-3D-GLRLM-RunPercentage feature in patients having survival > 5 years and < 5 years.


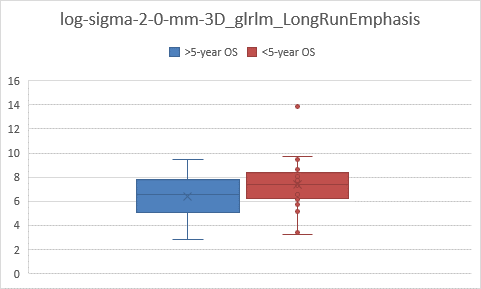


Supplementary figure 7: figure shows distinct distribution of LOG-sigma-2.o-mm-3D-GLRLM-LongRunEmphasis feature in patients having survival > 5 years and < 5 years.


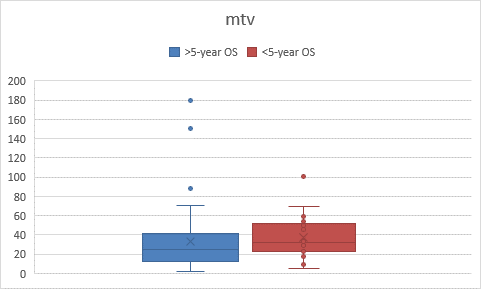


Supplementary figure 8: figure shows distribution of metabolic tumor volume (mtv) in patients having survival > 5 years and < 5 years.


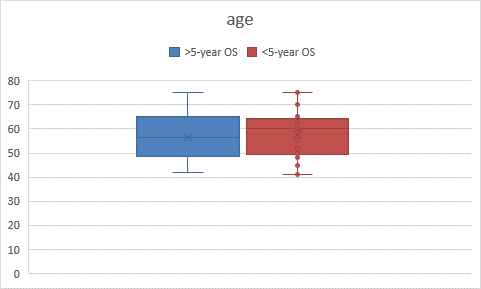


Supplementary figure 9: figure shows distribution of age of patients having survival > 5 years and < 5 years.

The following figures shows the AUC of all the prediction models in validation set.


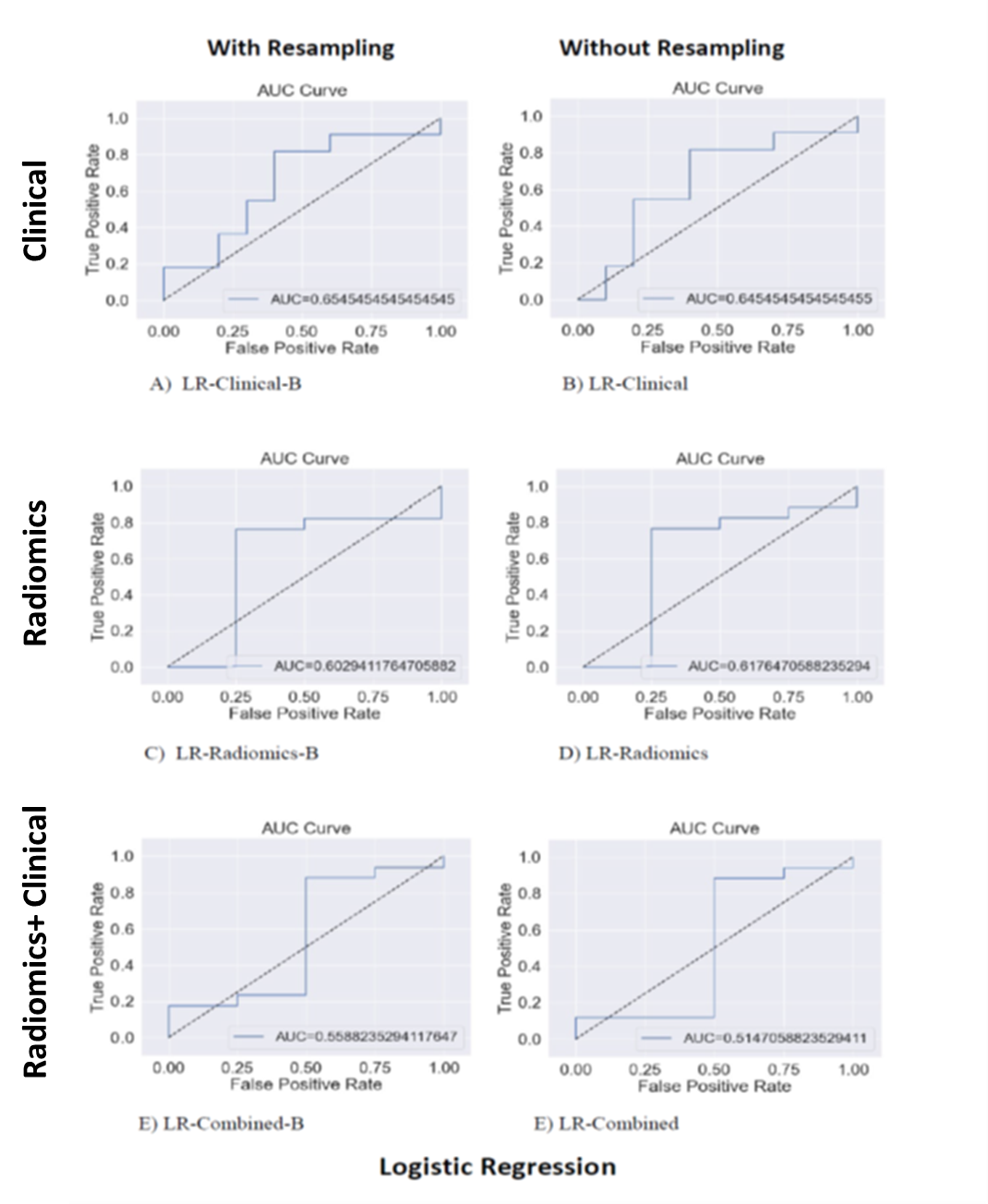


Supplementary figure 10: AUC curves of all the Logistic models are shown in this figure


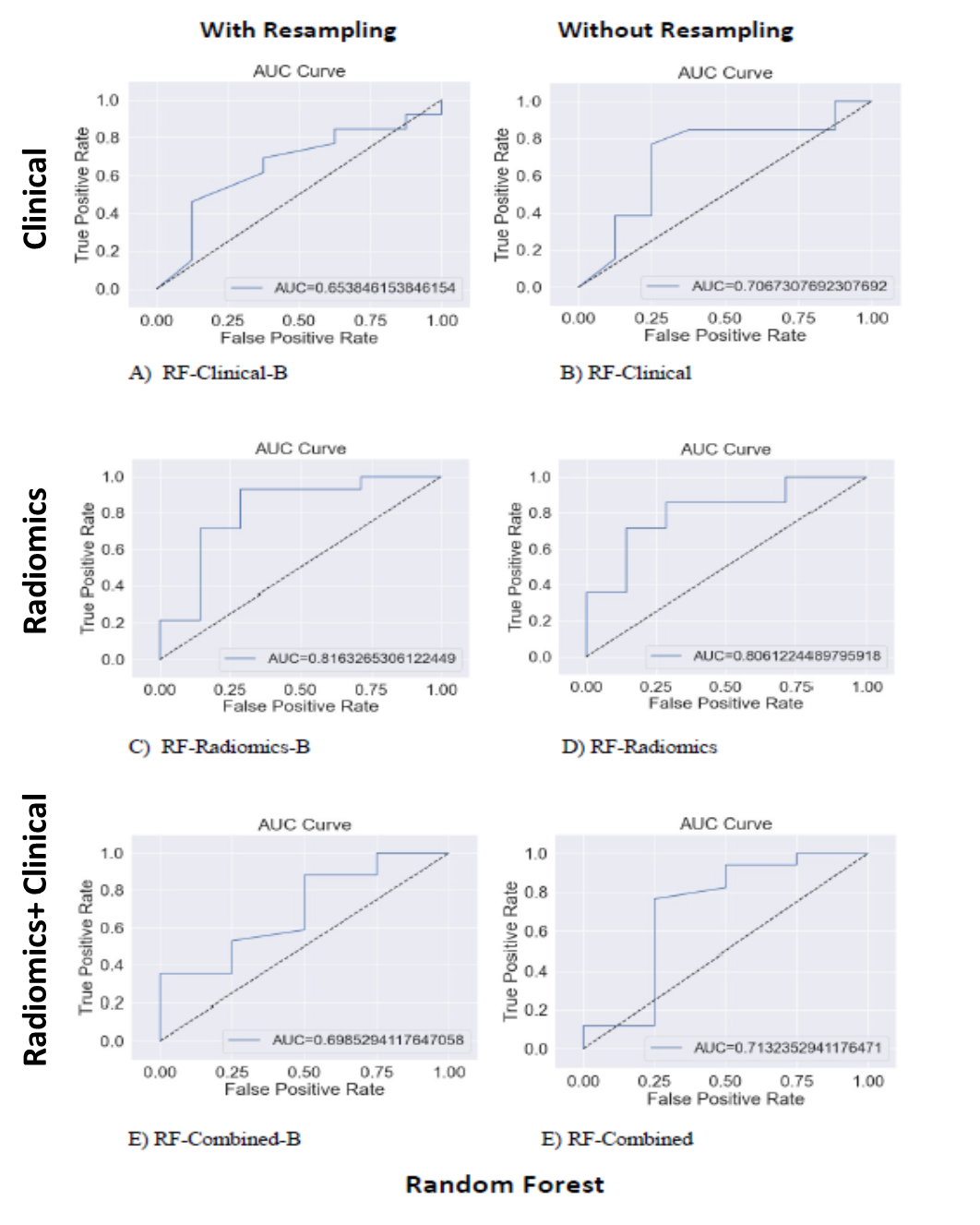


Supplementary figure 11: AUC curves of all the random forest models are shown in this figure


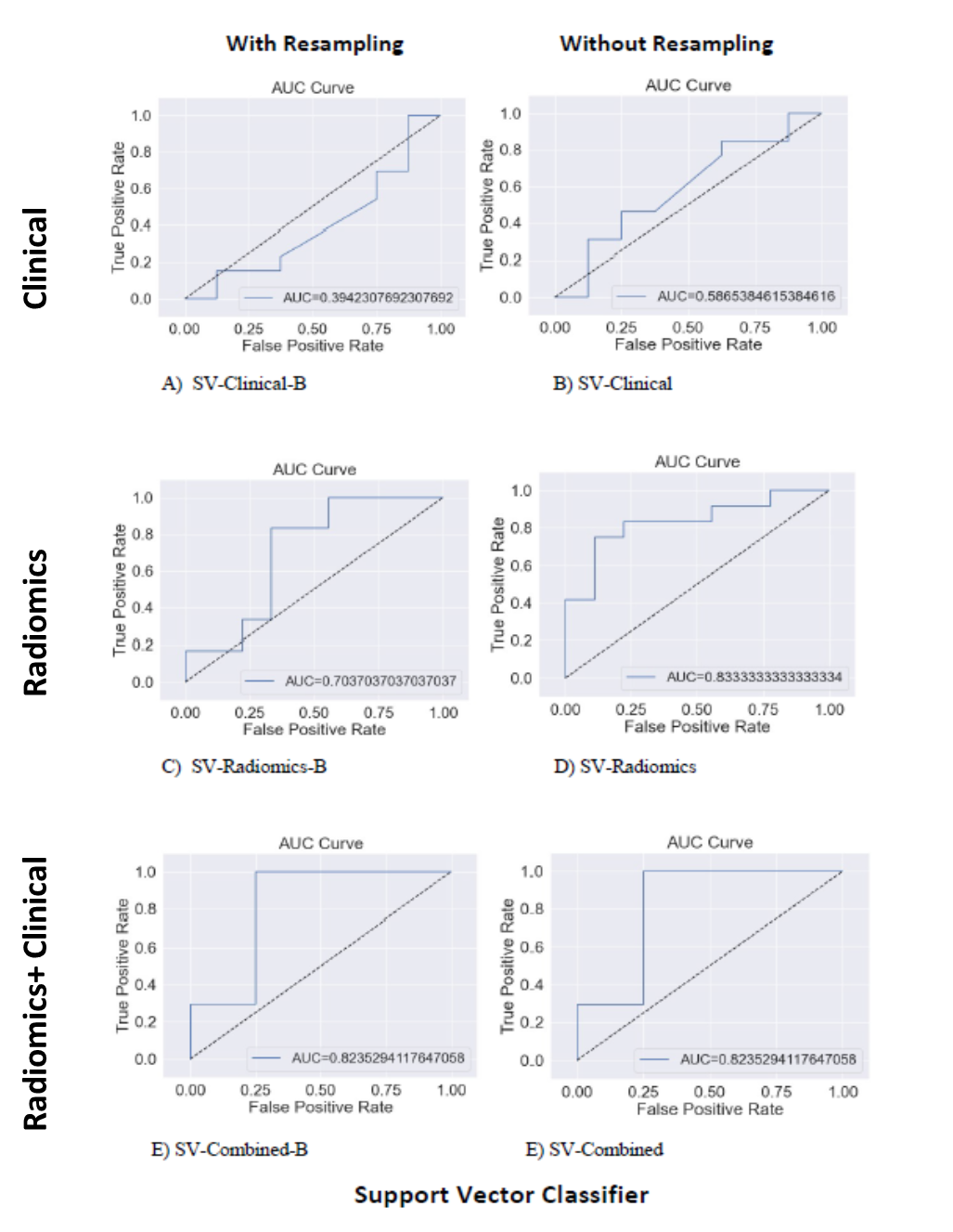


Supplementary figure 12: AUC curves of all the SVC models are shown in this figure


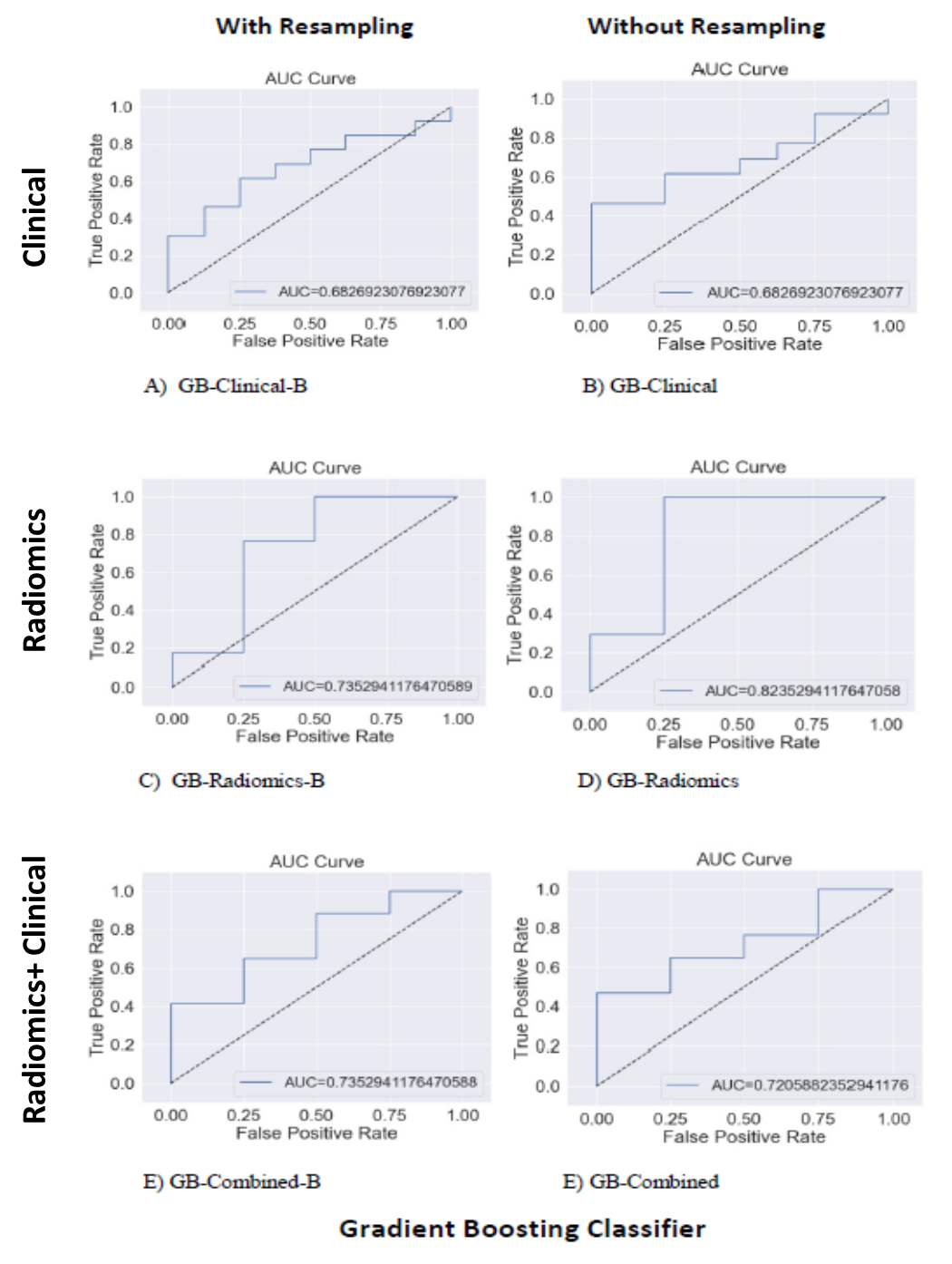


Supplementary figure 13: AUC curves of all the GBC models are shown in this figure
